# Connective tissue growth factor (CTGF) regulates the fusion of osteoclast precursors by inhibiting Bcl6 in periodontitis

**DOI:** 10.7150/ijms.41075

**Published:** 2020-02-24

**Authors:** YunJeong Choi, Ji Hyun Yoo, Jae-Hyung Lee, Youngkyun Lee, Moon-Kyoung Bae, Yong-Deok Kim, Hyung Joon Kim

**Affiliations:** 1 Department of Oral Physiology, BK21 PLUS Project, Periodontal Diseases Signaling Network Research Center, and Dental and Life Science Institute, School of Dentistry, Pusan National University, Yangsan, Republic of Korea, 50611; 2 Department of Maxillofacial Biomedical Engineering, School of Dentistry, Department of Life and Nanopharmaceutical Sciences, Kyung Hee Medical Science Institute, Kyung Hee University, Seoul, Republic of Korea, 02447; 3 Department of Biochemistry, School of Dentistry, Kyungpook National University, Daegu, Republic of Korea, 41940; 4 Department of Oral and Maxillofacial Surgery, Dental Research Institute, and Dental and Life Science Institute, School of Dentistry, Pusan National University, Yangsan, Republic of Korea, 50611

**Keywords:** CTGF, DC-STAMP, Bcl6, osteoclast fusion, periodontitis

## Abstract

Connective tissue growth factor (CTGF), an extracellular matrix protein with various biological functions, is known to be upregulated in multiple chronic diseases such as liver fibrosis and congestive heart failure, but the mechanism it undertakes to cause alveolar bone loss in periodontitis remains elusive. The present study therefore investigates the pathways involving CTGF in chronic periodontitis. RNA sequencing revealed a notable increase in the expression of CTGF in chronic periodontitis tissues. Also, TRAP staining, TRAP activity and bone resorption assays showed that osteoclast formation and function is significantly facilitated in CTGF-treated bone marrow-derived macrophages (BMMs). Interestingly, western blotting and immunofluorescence staining results displayed that CTGF had little effect on the osteoclastogenic differentiation mediated by the positive regulators of osteoclastogenesis such as nuclear factor of activated T cells 1 (NFATc1). However, following results showed that both the mRNA and protein expressions of B cell lymphoma 6 (Bcl6), a transcriptional repressor of “osteoclastic” genes, were significantly downregulated by CTGF treatment. Moreover, CTGF upregulated the expressions of v-ATPase V0 subunit d2 (ATP6v0d2) and Dendritic cell-specific transmembrane protein (DC-STAMP) which are osteoclastic genes specifically required for osteoclast cell-cell fusion in pre-osteoclasts. Findings from this study suggest that CTGF promotes the fusion of pre-osteoclasts by downregulating Bcl6 and subsequently increasing the expression of DC-STAMP in periodontitis. Understanding this novel mechanism that leads to increased osteoclastogenesis in periodontitis may be employed for the development of new therapeutic targets for preventing periodontitis-associated alveolar bone resorption.

## Introduction

Periodontitis is a chronic inflammatory disease initiated by the colonization of complex subgingival bacterial plaque biofilms 1, and there have been a wide range of host and microbial factors reported to contribute to alveolar bone loss, the hallmark of the disease. Although the complex mechanisms regulating bone resorption in periodontitis is not fully understood, previous studies have postulated that the prolonged and persistent osteoclastic activation in the periodontium is one of the main factors responsible for the alveolar bone loss in periodontitis 2, 3.

Bone resorption is a basic physiologic process mediated by osteoclasts that differentiate from monocyte/macrophage precursors under the regulation of critical cytokines such as macrophage colony stimulating factor (M-CSF), receptor activator of nuclear factor kappa-Β ligand (RANKL), and osteoprotegerin 4. As osteoclasts are the principal bone resorptive cells, local stimulation of their activity is an essential requirement for alveolar bone loss 5. In the context of periodontitis, it is now generally accepted that once the biologically active substances within bacterial plaque induce a local inflammatory response in the gingival soft tissues and periodontium 6, T- and B-cell-mediated host immune responses against bacterial components elicit the aberrant activation of osteoclasts based on the production of RANKL by activated lymphocytes 7.

Osteoclasts are multinucleated cells that arise as a result of cell fusion 8. Studies have shown that fusion is required for the maturation of osteoclasts and for gaining functional ability to resorb bone 9, 10. The essential cell-cell fusion of mononuclear macrophages to multinuclear osteoclasts is regulated by dendritic cell-specific transmembrane protein (DC-STAMP), a seven-transmembrane protein 10. Connective tissue growth factor (CTGF) is the second member of the CCN family of proteins (CCN2), and CCN family proteins are involved in a number of biological processes in development, tissue repair, and tumor suppression. In the skeletal system, mounting evidence have indicated that CTGF promotes the proliferation and maturation of chondrocytes and osteoblasts and induces excess osteoclastic functions 11. Results from a study using MDA231, a human breast cancer cell line, revealed that the osteolysis metastasis was decreased by treating CTGF-neutralizing antibody 12. CTGF was highly expressed in MDA231 cells, and the bone destruction induced by MDA231 cells may be due to the increased activity of the osteoclasts activated by CTGF 11. Moreover, another study reported that CTGF induced by tumor necrosis factor α (TNF- α) upregulates osteoclastogenesis in patients with rheumatoid arthritis 13.

In the present study, we hypothesized that CTGF may be enhancing osteoclastogenesis in periodontitis, consequently leading to the alveolar bone loss. Indeed, CTGF is evidenced to potentiate RANKL- induced osteoclastogenesis in its late stage in osteoclast precursor cell line RAW 264.7 cells 14 and in its early stage by binding to RANK in MC3T3‐E1, a mouse osteoblastic cell line 15. Here, we showed that CTGF causes Bcl6 downregulation, an effect that leads to increased DC-STAMP expression. B cell lymphoma 6 (Bcl6) was originally identified as a proto-oncogene because its chromosomal translocation and constitutive expression promotes lymphomagenesis 16. Previously, Miyauchi et al. have demonstrated that Bcl6 inhibits osteoclast differentiation by attenuation transcription of osteoclastic genes *in vitro* 17, but its mechanism involving CTGF that leads to enhanced osteoclastogenesis in periodontitis was unexplored. Therefore, we examined the effects of CTGF on the regulators of osteoclastogenesis.

## Materials and methods

### Patient recruitment and RNA sequencing of gingival tissue samples

Gingival tissue samples were collected from healthy patients or chronic periodontitis patients at Kyungpook National University Dental Hospital. Periodontitis-affected site had a probing depth of ≥4 mm, clinical attachment level of ≥4 mm, and displayed bleeding upon probing. Ten samples were obtained from 9 healthy patients and 10 from 4 chronic periodontitis patients. All patients were non-smoking, not associated with infection or autoimmune diseases at the time of sample collection, and did not have untreated metabolic or systemic diseases. The gingival biopsies were approximately 3 mm^2^ in size and collected from gingival margins. They were washed immediately with phosphate-buffered saline (PBS) and stored in RNAlater solution (Thermo Fisher Scientific, Waltham, MA, USA) at -70°C.

Total RNAs were extracted using mirVana RNA isolation kits (Thermo Fisher Scientific, Waltham, MA, USA) after lysing frozen tissues using a disposable pestle grinder system (Thermo Fisher Scientific, Waltham, MA, USA). mRNAs were purified using poly-T oligo-attached magnetic beads and RNAs of ~300 bp were isolated by gel electrophoresis. cDNA libraries were synthesized using Truseq RNA sample preparation kits (Illumina, San Diego, CA, USA) and amplified cDNAs were loaded and sequenced in paired-end (PE) sequencing mode using the HiSeq 2000 sequencing system (Illumina). To determine the significance of differences between groups, the *DESeq* package was used as previously described 18.

### Mice

All animal procedures were approved by the Pusan National University Institutional Animal Care and Use Committee (PNU-IACUC) and carried out according to the guidelines issued by the animal care committee of the Institute of Laboratory Animal Resources of Pusan National University (PNU-2019- 2200).

### Reagents

M-CSF and RANKL were purchased from PeproTech (Rocky Hill, NJ, USA). Primary antibodies against p-AKT, AKT, p-ERK, ERK, p-JNK, JNK, p-p38, and p38 were purchased from Cell Signaling (Beverly, MA, USA). HRP-conjugated secondary antibodies were acquired from (GenDEPOT, Barker, TX, USA). Anti-lamin B and anti-NFATc1 antibodies were obtained from Santa Cruz Biotechnology (Santa Cruz, CA, USA). DAPI and Cy3-conjugated secondary antibodies were acquired from Sigma-Aldrich (St. Louis, MO, USA). Keyhole-Limpet-Hemocyanin (KLH)-conjugated anti-DC-STAMP antibody and IgGκ light chain binding protein (m-IgGκ BP-PE) were from Santa Cruz Biotechnology (Millipore, Temecula, CA, USA).

### Osteoclast generation

Bone marrow was extracted from the femora and tibiae of 5-week-old female ICR mice and flushed with α-minimum essential medium (α-MEM; Welgene Inc., Deagu, Republic of Korea) using a syringe. Bone marrow cells were collected by centrifugation and incubated with red blood cell lysis buffer for 10 seconds at room temperature. After purification, cells were seeded in 48-well plates at a density of 4 × 10^4^ cells/well and cultured in α-MEM containing 10% fetal bovine serum (FBS), 100 U/ml penicillin, and 100 μg/ml streptomycin with M-CSF (30 ng/ml) for 2 days. After 2 days, cells were treated with RANKL (100 ng/ml) and different concentrations of CTGF. The adherent cells were used as osteoclast precursors (bone marrow-derived macrophages, BMMs). BMMs were cultured for 4 days after RANKL treatment and the media were changed every 2 days.

### Tartrate-resistant acid phosphatase (TRAP) staining and activity assay

Osteoclastic differentiation of BMMs was evaluated by TRAP staining using the Leukocyte Acid Phosphatase Kit (Sigma-Aldrich, St. Louis, MO, USA) and TRAP activity assay (TRAP Assay Kit; Takara, Shiga, Japan). Cultured cells were fixed in 3.7% paraformaldehyde for 10 minutes, treated with 0.1%Triton X-100 in PBS at room temperature for 5 minutes, and rinsed three times with deionized water. Finally, cells were incubated with 0.01% naphthol AS-MX phosphate and 0.05% fast red violet LB salt in 50mM of sodium tartrate and 90mM of sodium acetate (pH 5.0) for 1 hour at 37 ℃ and rinsed 3 times with deionized water. TRAP activity was measured by using the cell culture supernatant generated after staining.

### *In vitro* bone resorption assay

BMMs were seeded on sterilized dentin slices (Immunodiagnostic Systems Inc., Boldon, UK) placed in 48-well plates as previously described 19. Cells were cultured under three different conditions: the negative control was cultured with α-MEM containing 10 % FBS, 100 U/ml penicillin, and 100 μg/ml streptomycin and 30 ng/ml of M-CSF, the positive control was treated with 100 ng/ml RANKL in addition to the negative control, and the CTGF group was treated with 100 ng/ml of CTGF in addition to the positive control. After 7 days, each well was washed with deionized water. To measure the depth and area of resorption pits, each disc was observed and analyzed using Zeiss LSM 5 PASCAL laser-scanning microscope (Carl Zeiss Inc., Thornwood, NY, USA).

### Western blotting

For Western blot analysis, BMMs were grown in 6-well plates at a density of 3 × 10^5^ cells/well and treated with 100 ng/ml RANKL and different concentrations of CTGF. Cells were harvested after lysis in cold RIPA buffer (40 mM Tris-Cl, 10 mM EDTA, 120 mM NaCl, and 0.1 % NP-40) containing protease inhibitor cocktail (Sigma-Aldrich, St. Louis, MO, USA), homogenized by sonication, and centrifuged at 4 °C for 10 minutes at 14,000 rpm. The supernatant was collected and the protein concentrations of the samples were determined using the Smith assay. The membranes were denatured using 5× SDS-PAGE sample buffer (TransLab, Daejeon, Republic of Korea) and 50 μg/lane were loaded onto 10% SDS-PAGE gels. Resolved proteins were transferred to nitrocellulose membranes and non-specific sites were blocked by 5% skim milk in TBS-T for 1 hour. Nitrocellulose membranes were immunoblotted overnight at 4 °C with primary antibodies diluted 1:1000 in Tris buffer saline with Tween-20 (TBST; Sigma-Aldrich, St. Louis, MO, USA). After washing 4 times with TBST for 10 minutes, blots were incubated for 1 hour at room temperature with HRP-conjugated secondary antibodies diluted 1:5000. Proteins were visualized by means of an enhanced chemiluminescent detection reagents (SuperSignal West Pico PLUS Chemiluminescent Substrate; Thermo Fisher, Waltham, MA, USA).

### Immunofluorescence staining

BMMs were seeded in 48-well plates at a density of 4 × 10^4^ cells/well, plated on sterile glass coverslips, treated with RANL and different concentrations of CTGF for 2 days, fixed for 10 minutes with 3.7 % formaldehyde, permeabilized in 0.1 % Triton X-100, and blocked in 1% BSA in PBS. Cells were then incubated for 1 hour at room temperature with antibodies against lamin B and NFATc1 (1:1000) in 1% BSA solution. After washing with TBS, cells were incubated for 1 hour at room temperature with DAPI or Cy3-conjugated secondary antibodies, washed three times with TBS, and mounted on glass slides. Images were acquired by confocal microscopy (FV300; Olympus, Tokyo, Japan).

### RT-PCR

Total RNA was harvested from cultured BMMs using TRIsure™ (Bioline, London, UK) and cDNAs were synthesized from 3 μg of isolated RNAs using Superscript II Reverse Transcriptase (Invitrogen, Carlsbad, CA, USA). Amplification was performed with StepOnePlus Real-Time PCR System (Applied Biosystems, Life Technologies, Carlsbad, CA, USA).

### Real-time PCR

Real-time PCR was performed using SYBR Green Master Mix reagents (Kapa Biosystems, Woburn, MA, USA) and ABI 7500 unit (Applied Biosystems, Carlsbad, CA, USA). *HPRT* mRNA expression was used as an endogenous control. The sequences of the primers used are as follows: *HPRT* forward: 5′-CCTAAGATGATCGCAAGTTG-3′; *HPRT* reverse: 5′-CCACAGGGACTAGAACACCTGCTAA -3′; *V-ATPaseV0d2* forward: 5′AGTGCAGTGTGAGACCTTGG-3′; *V-ATPaseV0d2* reverse: 5′-CAAAGCAACAGACTCCCAAA-3′; *DC-STAMP* forward: 5′-GACCTTGGGCACCAGTATTT-3′; *DC-STAMP* reverse: 5′-CAAAGCAACAGACTCCCAAA-3′; *Bcl6* forward: 5′-ATGAGATTGCCCTGCATTTC-3′; *Bcl6* reverse: 5′-TTCTTCCAGTTGCAGGCTTT-3′. All reactions were run in triplicate.

### Surface antigen expression

BMMs were harvested for flow cytometry analysis of surface DC-STAMP expression. Cells were washed twice with ice cold PBS, stained with KLH-conjugated anti-DC-STAMP antibody for 30 minutes in the dark on ice, washed 3 times, and stained with m-IgGκ BP-PE for 30 min in the dark on ice. Cells were analyzed by BD FACS Cell Analyzer (BD Bioscience, Heidelberg, Germany) equipped with FACSuite software, v1.0.5.3841 (BD Bioscience).

### Statistical analysis

The results are presented as means ± standard deviations (SDs). Statistical significance was determined using the two-tailed Student's *t*-test and *p* values of < 0.05 or 0.01 -denoted as * and **, respectively -were regarded significant. All experiments were repeated three times.

## Results

### CTGF gene expression is upregulated in human periodontitis tissues

**Figure 1:**
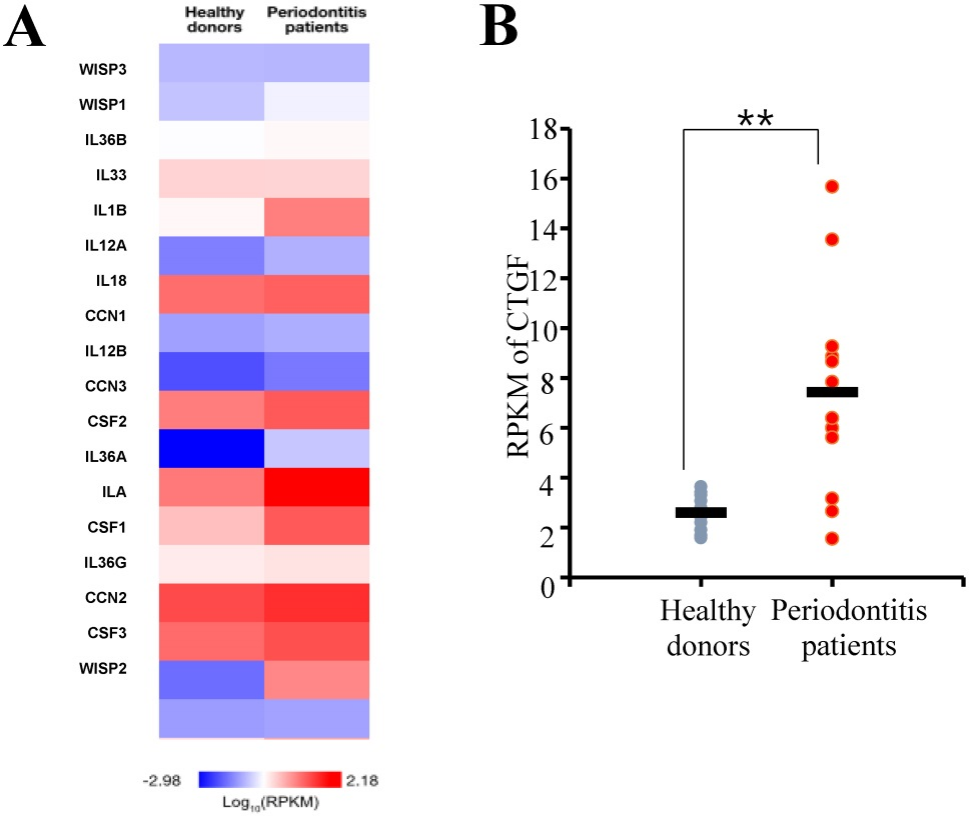
RNA sequencing analysis of periodontal tissues. (**A**) A Heat map of genes associated with inflammatory responses. The median gene expression of each gene from the samples was normalized by log_10_RPKM. RPKM, Reads per kilobase of exon per million mapped reads. (**B**) Connective tissue growth factor (CTGF) was found to be differentially expressed between the healthy and periodontitis gingival tissues. RPKM, Reads per kilobase of exon per million mapped reads. **p < 0.01.

The differential gene expression between the periodontitis-affected and healthy gingiva was evaluated by *DESeq* package as previously described 20. From the RNA sequencing assay, genes that showed differences in expressions greater than 2-fold were selected and a false discovery rate (FDR) of < 0.05 were applied. First, the expression levels of gene associated with inflammation responses were evaluated, and the result showed that a number of inflammatory cytokine genes were highly expressed in gingiva samples of periodontitis patient (Fig. 1A). Among the genes, CTGF was found to be upregulated significantly in the tissue samples of periodontitis patients (Fig. 1B).

### CTGF enhances osteoclast formation

**Figure 2:**
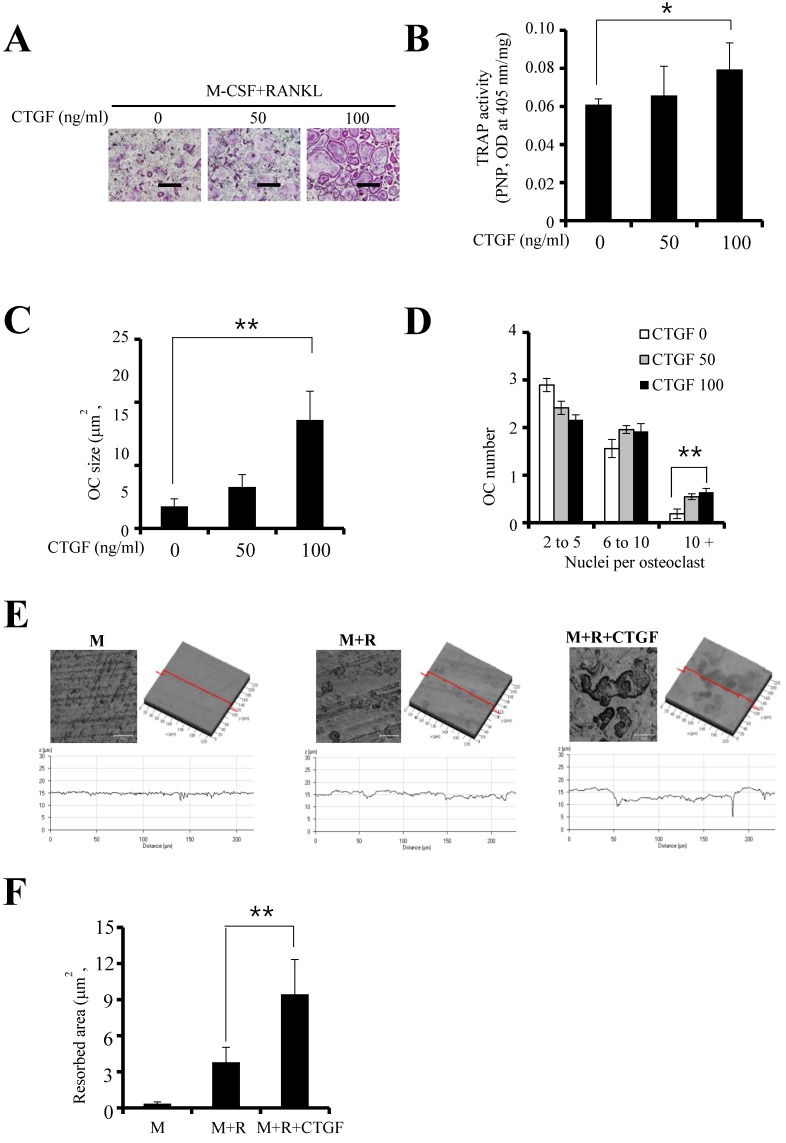
CTGF enhances osteoclast formation and function. (**A**) Bone marrow macrophages (BMMs) were treated with different concentrations of CTGF in the presence of 30 ng/ml M-CSF and 100 ng/ml RANKL. Cultured BMMs were stained with tartrate-resistant acid phosphatase (TRAP) and examined under a light microscope. Scale bar, 200 μm. (**B**) Relative TRAP intensities were measured after 4 days of culture. (**C, D**) The size and number of OCs were also measured by TRAP staining. (**E**) Resorption pits on dentin discs were visualized using a confocal laser scanning microscope. Dark areas indicate the resorbed surfaces. Scale bar, 200 μm. (**F**) Relative resorption areas were measured in four randomly selected images from each bone resorption assay (M: M-CSF, R: RANKL). All experiments were repeated three times. *p < 0.05, ** *p* < 0.05.

To investigate the effect of CTGF on RANKL- stimulated osteoclastogenesis, TRAP staining and TRAP activity assay were performed. Results showed that CTGF enhanced osteoclastogenesis as well as the expression of its cytochemical marker in a concentration-dependent manner (Fig. 2A and B). Moreover, increasing CTGF concentrations resulted in increased size and number of osteoclasts containing more than 10 nuclei (Fig. 2C and D). When the BMMs were treated with 100 ng/ml CTGF, the depth (Fig. 2E) and area (Fig. 2F) of the dentin samples resorbed by the differentiated osteoclasts increased significantly. These results indicate that CTGF enhances osteoclastogenesis and osteoclast function induced by M-CSF and RANKL.

### CTGF does not affect the expression of pro-osteoclastogenic factors

**Figure 3:**
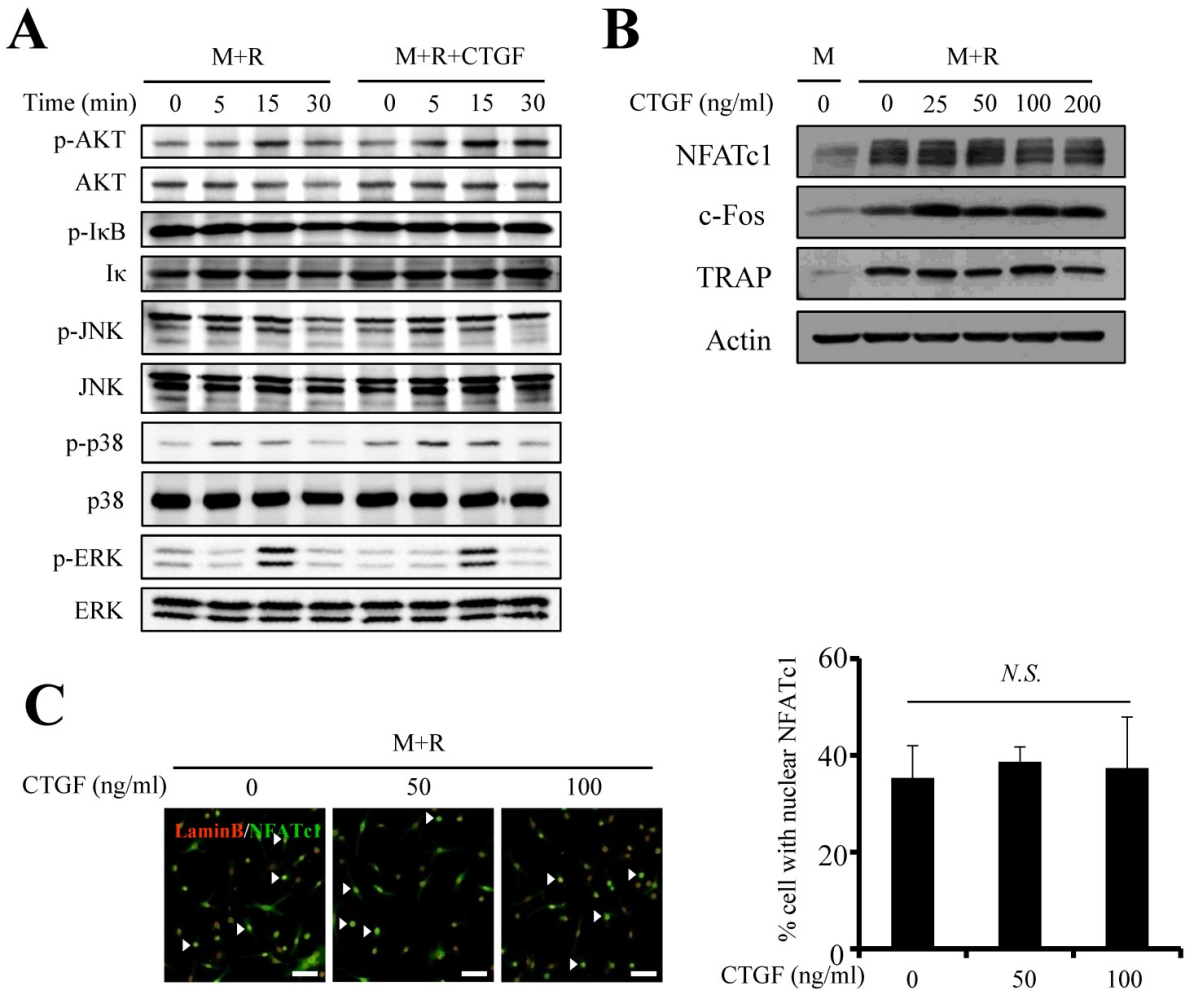
CTGF has little effect on the pro-osteoclastogenic signaling pathways. (**A**) MAPK and NF-κB pathways were examined by western blotting using the indicated antibodies. (**B**) Protein levels of pro-osteoclastogenic-transcription factors (NFATc1 and c-Fos) and osteoclastogenic differentiation marker (TRAP) were examined. (**C**) Nuclear translocation of NFATc1 was evaluated by immunofluorescence staining with anti-NFATc1 (FITC-labeled, green) and anti-lamin B (Cy3-labeled, red) antibodies. Numbers of nuclei stained for NFATc1 (arrows) were counted (M: M-CSF, R: RANKL). All experiments were repeated three times. Scale bar, 50 μm. N.S., not significant.

Next, the impact of CTGF treatment on RANKL-induced osteoclast differentiation was assessed by examining expression levels of key modulators in mitogen-activated kinase (MAPK) and Nuclear factor-κB (NF-κB) pathways. The protein expressions of p-IκB, p-JNK, p-ERK, well-known differentiation factors of osteoclastogenesis, were unchanged whether CTGF was treated or not (Fig. 3A). However, the phosphorylations of AKT and p38 showed slight increases upon 100 ng/ml of CTGF treatment. Interestingly, CTGF treatment did not affect the expressions of c-fos, an essential pro-osteoclastogenesis factor expressed during the early stages of osteoclastogenesis, and nuclear factor of activated T cells 1 (NFATc1), the master regulator of osteoclastogenesis (Fig. 3B). Expression levels of TRAP, an important differentiation marker of osteoclastogenesis, was uninfluenced as well. In support of the Western blotting results, immunofluorescence results showed that CTGF had no effect on the nuclear translocation of NFATc1 (Fig. 3C). These data reveal that CTGF does not directly induce osteoclastogenesis by upregulating the positive regulators of RANKL-induced osteoclast differentiation.

### CTGF indirectly induces osteoclastogenesis by suppressing anti-osteoclastogenic factor expression

**Figure 4:**
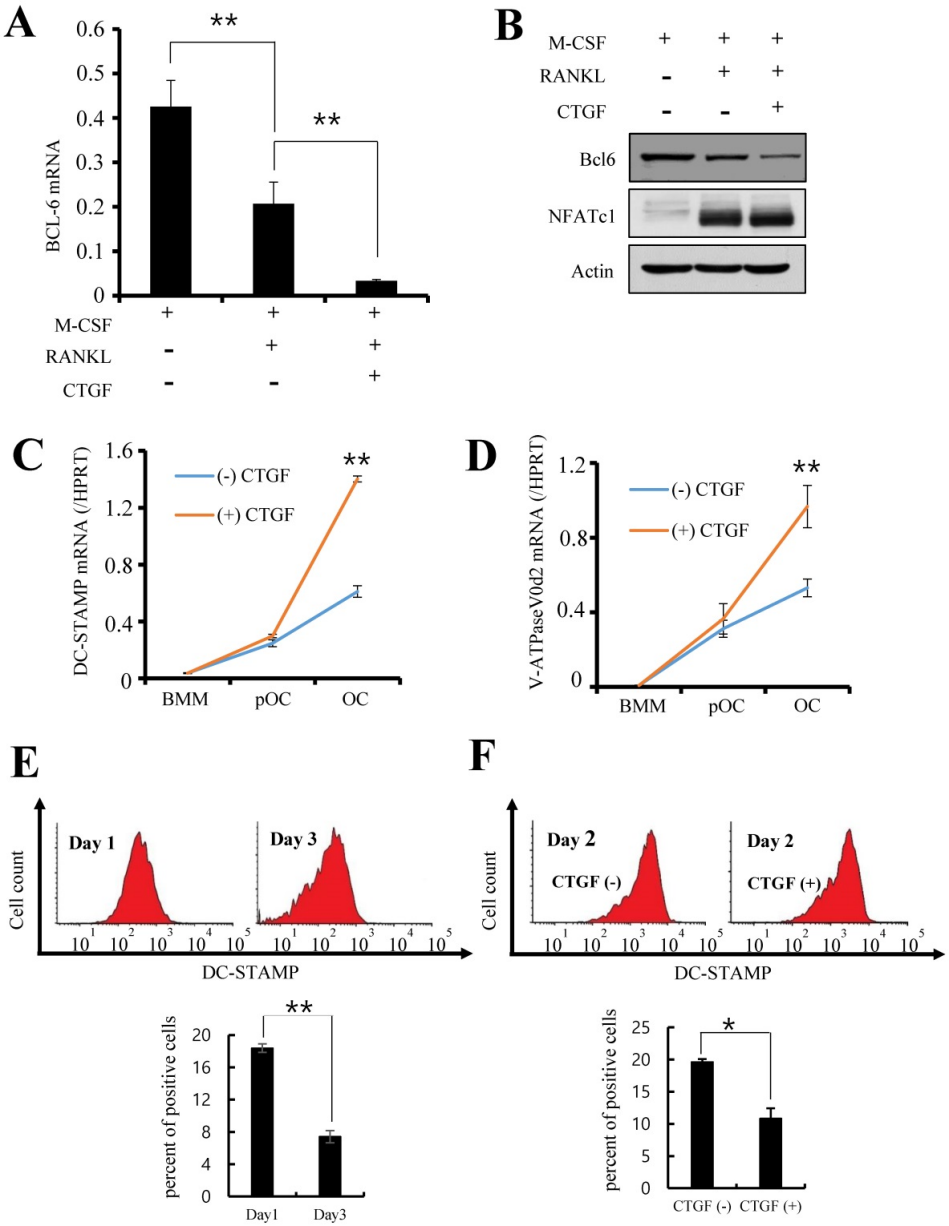
CTGF facilitates the multinucleation and maturation of osteoclasts by inhibiting Bcl6 expression and inducing the expressions of DC-STAMP and ATP6v0d2. (**A**) The mRNA expression of *Bcl*-6 in osteoclasts was evaluated by real-time PCR, and (**B**) the protein expressions of Bcl-6 and NFATc1 in osteoclasts treated with 100 ng/ml CTGF were analyzed by western blotting. ** *p* < 0.01 (**C, D**) mRNA expressions of *DC-STAMP* and *ATP6v0d2* in BMMs treated with or without 100 ng/ml CTGF determined by real-time PCR. BMMs were cultured for different days: day 0 (BMM), day 2 (pOC), and day 3 (OC). BMM, bone marrow macrophage; pOC; pre-osteoclast; OC; osteoclast. (**E**) Expressions of DC-STAMP on the surface of BMMs treated with or without 100 ng/ml CTGF were examined on day 1 and 3 by flow cytometry. (**F**) The ratio of pre-osteoclasts with a fluorescence intensity exceeding the threshold value (10^3^) was calculated. All experiments were repeated three times. ** *p* < 0.01.

Since CTGF did not have an effect on key inducers of osteoclastogenesis, suppressors of the process were investigated. BMMs were cultured with RANKL and 100 ng/ml of CTGF and the expression levels of B cell lymphoma 6 (Bcl6), a transcriptional repressor of osteoclast differentiation, were observed. As shown in Fig. 4A, the mRNA expression of Bcl6 was significantly suppressed by CTGF treatment. Moreover, its protein expression was markedly downregulated in BMMs treated with CTGF compared to that in the control (Fig. 4B). Thus, CTGF treatment has no significant effect on NFATc1 expression but downregulates Bcl6 in order to facilitate osteoclastogenesis under RANKL stimulation.

### CTGF accelerates cell-to-cell fusion of osteoclast precursors

Next, the downstream factors of the Bcl6 pathway were examined to identify the mechanism underlying the accelerated osteoclastogenesis by CTGF. BMMs were treated with RANKL and 100 ng/ml of CTGF for 0, 2, or 3 days and the mRNA levels of DC-STAMP and v-ATPase V0 subunit d2 (ATP6v0d2) were measured. The promoter region of DC-STAMP is a direct binding target of Bcl6 21, and both DC-STAMP and ATP6v0d2 are known to regulate the cell-cell fusion of osteoclast precursors 22, 23. The results show that the expression levels of the two genes were significantly increased in the CTGF-treated pre-osteoclasts compared to the control (Fig. 4C and D).

**Figure 5:**
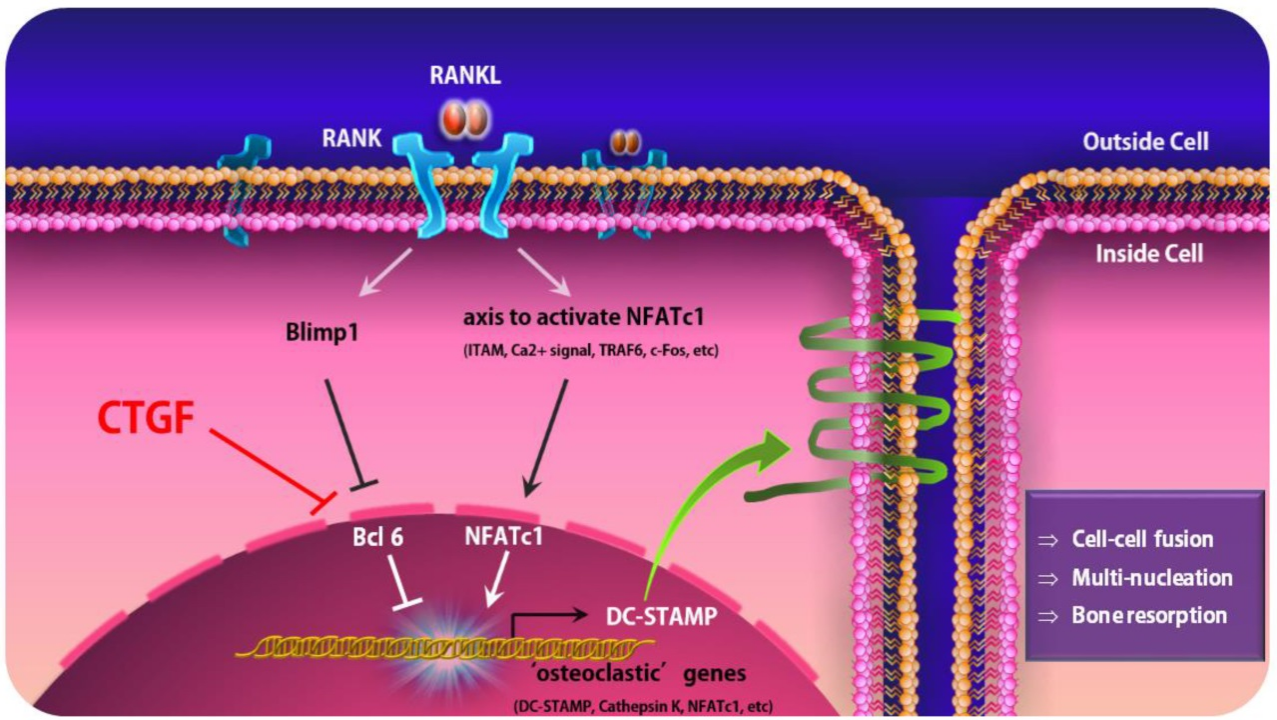
A schematic model of osteoclastogenesis regulated by CTGF that leads to the activation of DC-STAMP. Under normal circumstances, RANKL-RANK interaction leads the activation of NFATc1, a crucial positive regulator for osteoclastogenesis, and Blimp1-Bcl-6, the negative regulators, to regulate osteoclastogenesis and bone homeostasis. When CTGF is upregulated, it suppresses the expression of Bcl6 and induces the dissociation of Bcl-6 from osteoclastic gene promoters, leading to the overexpression of DC-STAMP.

An earlier study has reported that DC-STAMP migrates from the extracellular surface to the cytoplasm during the fusion and maturation of osteoclasts and therefore its surface expression decreases gradually as mature, multinucleated osteoclasts form during osteoclastogenesis 24. To show that CTGF is involved in this migration process, the surface expression of DC-STAMP during osteoclastogenesis was observed by flow cytometry. In support of previous data by Chiu et al. 24, DC-STAMP surface expression on culture day 3 was significantly lower than that on day 1 (Fig. 4E). In addition, BMMs treated with CTGF showed markedly lower surface expressions of DC-STAMP compared to the control on day 2 when BMMs are at the pre-osteoclast stage and DC-STAMP expression is supposedly at its peak (Fig. 4F). These results suggest that CTGF may be enhancing osteoclast fusion and maturation by accelerating the DC-STAMP-mediated fusion of pre-osteoclasts (Fig. 5).

## Discussion

CTGF is a cysteine-rich secretory protein containing four conservative modules which are IGFBP module, VWC type C module, TSP type 1 repeat, and C-terminal module 25. Early studies have focused on its involvement in fibrosis because CTGF had been shown to promote the proliferation, migration, and adhesion of fibroblasts by inducing TGF-beta 26-31. However, CTGF has recently attracted interest due to its role in oral diseases. For example, increased expressions of CTGF have been documented in lesions caused by phenytoin, an anti-seizure medication, nifedipine, an anti-hypertensive drug, and ciclosporin, an immune suppressor 32. These drugs from medical therapy ultimately cause tissue-specific gingival overgrowth which presents major problems for maintaining oral hygiene due to increased risk for infection and inflammatory complications, difficulty with mastication, and a disfigured appearance accompanied by the swelling of gingiva. Interestingly, CTGF upregulation in the fibroblasts of gingival tissues have altered signal transduction pathways that confer unexpected resistance to effects of some inflammatory mediators, which are considered to contribute to the tissue specificity and fibrosis 32.

Unfortunately, little information is available on the precise role of CTGF in regard to periodontitis and bone destruction. Early diagnostic study with 21 individuals with or without periodontitis found for the first time the expressions of CTGF along with TGFβ1 mRNA are increased and correlated in patients with periodontitis 33. Authors from this study suggested from this result that both tissue destruction and tissue regeneration must co-exist in periodontitis. It is compelling to note that although CTGF is involved, the tissue-regenerative process is dominant in drug-induced gingival overgrowth while gingival tissue inflammation and destruction are predominant in periodontitis. In addition, our colleagues recently suggested that CTGF is significantly overexpressed in periodontitis tissues via RNA sequencing analysis 20. In the present study, our intention was to expand our understanding of the mechanism behind the upregulation of CTGF expression in periodontitis which leads to alveolar bone loss in the end.

Controlling osteoclastogenesis is critical for maintaining physiological bone homeostasis and preventing skeletal disorders, and there are several well-known regulators of osteoclastogenesis. When RANKL binds to its receptor RANK on osteoclast precursors, a broad range of signaling cascades including the canonical and non-canonical NF-κB pathways and MAPK pathways are facilitated, leading to the activation of activator protein-1 (AP-1; c-Fos and c-Jun) and cyclic-AMP response element binding (CREB) transcription factors. In addition, calcium signaling is induced, followed by the expression of key transcription factors such as Blimp1 and NFATc1 for osteoclast differentiation 34-37.

NFATc1 is the master regulator of osteoclastogenesis under RANKL stimulation, responsible for the regulation of genes related to osteoclast function as well as numerous genes non-essential to osteoclast function 38, 39. Positive regulators such as NFATc1 induce the expression of “osteoclastic” molecules essential for the differentiation and function of osteoclasts. Few of the key osteoclastic genes are: DC-STAMP, which is known to facilitate cell-cell fusion, cathepsin K (Ctsk) which promotes bone matrix proteolysis 40, 41, and NFATc1 which drives differentiation. On the other hand, the Blimp1-Bcl6 axis is a crucial negative regulator of osteoclast differentiation 10. B lymphocyte-induced maturation protein 1 (Blimp1) is a transcriptional repressor that inhibits the expression of Bcl6, likely by binding directly to the Bcl6 promoter, and Bcl6 is also a transcriptional repressor that antagonizes NFATc1 function 21. The expression of Bcl6 and Blimp1 is reciprocal in normal osteoclast formation, and the suppression of Bcl6 during osteoclast differentiation is required for the appropriate osteoclastogenesis and regulation of bone homeostasis 21.

In the present study, we discovered that CTGF treatment on BMMs did not affect the protein expressions of key inducers of osteoclastogenesis. Akt and ERK are signaling pathways activated by the binding of M-CSF to its receptor c-Fms, and p-JNK, p-p38, and p-IκB are downstream signaling pathways activated by c-Fos and NFATc1 expression in osteoclast precursors 42. However, our immunoblotting results demonstrated a slight increase in the expressions of p -AKT and p-ERK, and we predict that CTGF exerts a partial effect on osteoclast differentiation via a mechanism yet to be discovered. Next, we asked if the upstream signaling pathway that regulates NFATc1 expression was affected, and there was no change in the levels of c-Fos in cell cultures with CTGF. Finally, we shed light on the Bcl6-osteoclastic gene axis which is recognized as a force of negative regulation of osteoclast differentiation to study the pathway CTGF undertakes in periodontitis. Balanced osteoclast differentiation is precisely controlled and maintained by complex mechanisms at various levels, and accumulating evidence propose that RANK needs to overcome the transcriptional repressors expressed constitutively in osteoclast precursors in addition to activating positive signaling pathways for osteoclastogenesis 21. Therefore, it is becoming clearer that negative regulation of osteoclastogenesis and bone resorption plays a key role in bone homeostasis fine tuning bone remodeling and restraining excessive bone resorption in inflammatory settings. Our results revealed that CTGF significantly downregulated the mRNA and protein expressions of Bcl6 in osteoclasts. Taken together, our findings propose a model whereby the overexpression of CTGF in chronic periodontitis tissues induces the suppression of Bcl6, which results in hyper-osteoclast differentiation and subsequent loss of bone mass.

## Conclusions

In summary, it has been established that CTGF promotes osteoclastogenesis by inducing DC-STAMP expression in the late stage of osteoclastogenesis 11, but the processes leading to such events were elusive. We report for the first time that CTGF treatment does not affect the expression of NFATc1 but dramatically decreases Bcl-6 expression at the mRNA and protein levels, which in turn leads to the upregulation of DC-STAMP. Understanding these mechanisms provides new molecular targets since inhibition of NFATc1 has failed to increase bone mass but rather reduced bone mass 10. CTGF as well as the Blimp1-Bcl6 axis may become new therapeutics targets for preventing periodontitis-associated bone resorption.
